# Does the Prevalence of Obesity Affect the Demand for Soft Drinks? Evidence from Cross-Country Panel Data

**DOI:** 10.3390/ijerph19020938

**Published:** 2022-01-14

**Authors:** Fabrizio Ferretti, Michele Mariani, Elena Sarti

**Affiliations:** Department of Communication and Economics, School of Social Sciences, University of Modena and Reggio Emilia, 42121 Reggio Emilia, Italy; michele.mariani@unimore.it (M.M.); elena.sarti@unimore.it (E.S.)

**Keywords:** demand, obesity, obesogenic environment, socio-economic factors, soft drinks, unhealthy diet

## Abstract

The impact of soft drinks on obesity has been widely investigated during the last decades. Conversely, the role of obesity as a factor influencing the demand for soft drinks remains largely unexplored. However, understanding potential changes in the demand for soft drinks, as a result of changes in the spread of obesity, may be useful to better design a comprehensive strategy to curb soft drink consumption. In this paper, we aim to answer the following research question: Does the prevalence of obesity affect the demand for soft drinks? For this purpose, we collected data in a sample of 97 countries worldwide for the period 2005–2019. To deal with problems of reverse causality, an instrumental variable approach and a two-stage least squares method were used to estimate the impact of the age-standardized obesity rate on the market demand for soft drinks. After controlling for several demographic and socio-economic confounding factors, we found that a one percent increase in the prevalence of obesity increases the consumption of soft drinks and carbonated soft drinks by about 2.37 and 1.11 L per person/year, respectively. Our findings corroborate the idea that the development of an obesogenic food environment is a self-sustaining process, in which obesity and unhealthy lifestyles reinforce each other, and further support the need for an integrated approach to curb soft drink consumption by combining sugar taxes with bans, regulations, and nutrition education programs.

## 1. Introduction

The impact of soft drink consumption on obesity has been widely investigated during the last decades. The cumulative evidence from observational studies and experimental trials indicates that the regular consumption of soft drinks, particularly sugar-sweetened beverages (SSBs), causes unhealthy weight gains [1,2,3]. In contrast, the role of obesity as a factor influencing the demand for soft drinks remains largely unexplored. Most attempts to estimate the demand for soft drinks focus on measuring the various types of beverages own- and cross-price elasticities [4,5,6]. Although valuable for many purposes, especially for evaluating the effectiveness of ‘sugar taxes’, these approaches pay little or no attention to weight status as a determinant of soft drinks consumption. However, there is evidence of a feedback loop between the consumption of soft drinks and the prevalence of obesity [7]. Furthermore, understanding potential changes in the market demand for soft drinks, due to changes in the prevalence of obesity, may be helpful to design the role of taxation within a comprehensive strategy for tackling the current ‘obesity epidemic’ [8].

Although it is a complex and multifactorial condition, it is widely acknowledged that obesity would be largely preventable through ‘relatively simple’ lifestyle changes [9,10,11]. However, unhealthy lifestyles are greatly affected by the pervasive development of the so-called obesogenic environments [12]. People’s exposure to the set of physical, cultural, and socio-economic factors that promote excessive energy intake (and sedentary behavior) has been dramatically increasing during recent decades [13]. Soft drinks are ubiquitous in modern food systems, and they are a typical component of obesity-prone environments [14]. The vast majority of these non-alcoholic carbonated and non-carbonated beverages are low in nutrients but high in free sugars (added by the manufacturer, usually in the form of refined beet and cane sugar or by using high-fructose corn syrup) [15]. These sugary drinks provide empty calories [16] and are complement-in-consumption of many ultra-processed and unhealthy energy-dense foods [17].

**Figure 1:**
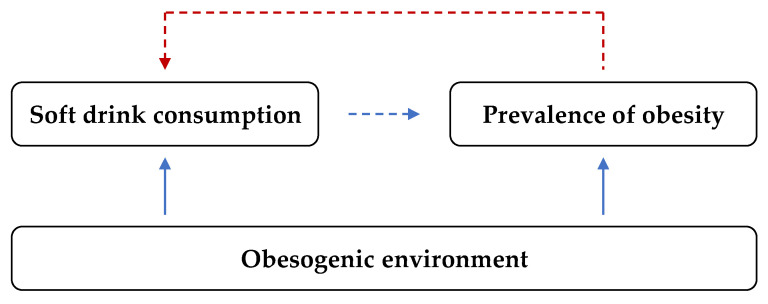
Interplay between soft drinks and obesity.

Obesity is a long-term chronic condition. People living with obesity usually face barriers in changing their eating habits towards healthy diets, such as lack of willpower, time constraints, and the pervasive availability of unhealthy foods and beverages [18]. Thus, one might ask whether the prevalence of obesity affects the market demand for soft drinks, that is, as briefly outlined in Figure 1, whether increases in the prevalence of obesity among the population increase the country’s demand for soft drinks, reinforcing the impact of soft drinks on the spread of obesity.

This paper estimates the market demand for soft drinks by explicitly including the prevalence of obesity as an explanatory variable. We aim to determine whether and how much the spread of obesity affects the demand for soft drinks. To this end, we collected data on per capita soft drink consumption, the prevalence rate of obesity, and some demographic and socio-economic control variables, in a sample of 97 countries for the period 2005–2019. To deal with endogeneity problems due to reverse causality between consumption and obesity, we used an instrumental variable (IV) approach and a two-stage least squares (2SLS) regression method.

## 2. Materials and Methods

### 2.1. Data

A demand function relates the quantity consumed of a given good to its price, the prices of related goods (i.e., complements and substitutes), the disposable income, and other ‘demand shifters’ variables. Data on the consumption and prices of soft drinks were obtained from Passport, Euromonitor’s Global Market Information Database [19].

From this database we retrieved information about the volume and value of soft drink sales in 99 countries worldwide. The quantity consumed (*QSD*) was computed dividing by the country total population both the on-trade and off-trade sales in volume of domestically manufactured and imported soft drinks. *QSD*, expressed in liters per person and per year, measures the per capita consumption of carbonated (i.e., regular and diet sodas) and non-carbonated (i.e., fruit juices, ready-to-drink tea, and coffee, as well as sports/energy and Asian drinks) beverages. In the same way, we also computed the consumption per capita for the sole sub-category of carbonated soft drinks (*QCA*). The population data used in these calculations were taken from the World Bank Open Data repository [20].

The market price of soft drinks (*PSD*) in each country was calculated as the ratio of total sales in value to total sales in volume. These average annual prices were converted from local currency to international dollars. To this end, we used the purchasing power parity (PPP) conversion factors, provided by the World Bank [21] within the International Comparison Program database. *PSD* measures the average retail selling price per liter, and it is expressed in 2017 constant prices. The same procedure was followed to compute the price of carbonated and non-carbonated soft drinks (denoted *PCA* and *PNC*, respectively) and the price of bottled (carbonated and still) water (*PWA*). Finally, data on Gross National Income per capita (GNI), measured in constant 2017 international dollars, collected from the World Bank Open Data repository [20], were included in the dataset to account for differences in consumers’ income.

The soft drink industry is dominated by a few firms that ‘think globally and act locally’ to expand soft drink consumption [22]. We considered the role of globalization in shaping modern food environments [23,24,25] by including among the determinants of the demand for soft drinks the KOF Index of economic globalization [26], developed by the Swiss Economic Institute as a measure of the degree of economic globalization (*GLO*), (i.e., international flows of goods, capital, and services).

In order to adjust for other potential confounding factors that can affect the demand for soft drinks, we also controlled for two more (demographic and economic) variables. The demographic structure of a population is, in fact, another key determinant of its dietary pattern. Soft drinks are popular beverages, especially among children, adolescents, and young adults. Furthermore, the soft drink industry usually targets young people as potential consumers [27] with aggressive marketing strategies on traditional and new media [28]. Therefore, we included the share of elderly people (*AGE*), measured by the number of people aged 65 and above as a percentage of the total population, as a determinant of soft drink consumption (the expected sign of the variable named *AGE* is thus negative). Similarly, the weight of agriculture in the economy (*AGR*), as measured by the share of agricultural value-added on the total value-added, was used to account for the level of development of the food system. In a traditional (i.e., non-industrialized) food system—characterized by a dominance of the agricultural sector, unorganized supply chains, and limited market infrastructure—environmental, cultural, and socio-economic barriers usually hamper the consumption of ultra-processed foods and beverages. Data on *AGE* and *AGR* were obtained from the World Bank Open Data repository [20].

Finally, data on the prevalence of obesity (*OBE*) were collected from the Global Health Observatory [29] of the World Health Organization (WHO). Specifically, *OBE* indicates the age-standardized prevalence of obesity, measured by the percentage of adults (aged 18+ years) who have a body mass index (BMI) equal to or greater than 30 kg/m^2^. However, because of the reverse effect of soft drinks on the prevalence of obesity, *OBE* is an endogenous variable in this demand model. As a result, the estimates obtained with ordinary least squares (OLS) regression would be biased by endogeneity (i.e., the correlation between an explanatory variable and the error term).

In order to obtain unbiased estimates of the impact of obesity on the consumption of soft drinks, we included in the dataset three factors, as instrumental variables, that previous research has linked to the obesity epidemic [13,30]. The first instrument was the average number of calories available for human consumption. Increased food energy supply has been proven to be a key driver of the worldwide spread of obesity [31]. This factor was captured by the dietary energy supply (*DES*), expressed in kcal per person per day, collected from FAOSTAT [32], the statistical database of the Food and Agriculture Organization (FAO) of the United Nation. Economic and social structural changes, such as the rise of the ‘service economy’ and the increasing number of people who live in urban areas, typically lead to more sedentary lifestyles and decreasing daily home and occupational energy-related expenditure [33]. The other two instrumental variables were thus the employment in the service sector, as a percentage of total employment (*EMP*) and the share of urban to total population (*URB*). Both variables were collected from the World Bank Open Data repository [20].

Overall, we gathered data on 99 countries. However, due to the limited availability of information on several variables, two regions (Hong Kong and Taiwan) were excluded from the regression analysis. The final dataset is a balanced panel data that contains information for 97 countries during the period 2005–2019 (for a total of 1455 observations). Table 1 provides basic descriptive statistics and a short description of each variable utilized in the study. A list of the countries included in the study is shown in Table A1 in Appendix A. Finally, the full dataset is available at the Mendeley Data repository (https://doi.org/10.17632/hkm25rbpsc.2, Accessed on 15 October 2020), and it is also collected in the Supplementary Material (Supporting Information File S1—Dataset).

### 2.2. Methods

In a regression model, the problem of endogeneity can stem from various issues, including simultaneity (i.e., when the explanatory and the explained variables influence each other at the same time), as in the case of obesity and consumption in the demand for soft drinks. The 2SLS is the most common IV regression method used to solve endogeneity problems [34]. Provided that suitable (i.e., relevant and exogenous) instruments are available, the endogenous variable is regressed in the first stage on the chosen instruments to isolate the component of its variation that is uncorrelated with the error term. In the second stage, this problem-free component is used to obtain unbiased estimates of the impact of the endogenous variable on its outcome. Specifically, in the second stage, we estimated a demand equation with the following fixed effects regression model:

(1) $${\mathrm{QSD}}_{\mathrm{it}}=\beta_{0}+\beta_{1}{\hat{OBE}}_{\mathrm{it}}+\beta_{2}{\mathrm{PSD}}_{\mathrm{it}}+\beta_{3}{\mathrm{GNI}}_{\mathrm{it}}+\beta_{4}{\mathrm{PWA}}_{\mathrm{it}}+\beta_{5}{\mathrm{GLO}}_{\mathrm{it}}+\beta_{6}{\mathrm{AGE}}_{\mathrm{it}}+\beta_{7}{\mathrm{AGR}}_{\mathrm{it}}+\alpha_{i}+u_{\mathrm{it}}$$

in which *OBE* is replaced by its predicted value ($\hat{OBE}$) from the first stage, and *α_i_* is the time-invariant country-specific constant (i.e., the country fixed effects representing unobserved heterogeneity). The subscripts *i* and *t* refer to the country and year, respectively. Because several cultural and social unobserved factors affect the demand for soft drinks [35,36], a fixed-effects regression model was used to control for potential country-specific omitted variables, assuming that these factors remained relatively constant throughout the period considered.

**Figure A1:**
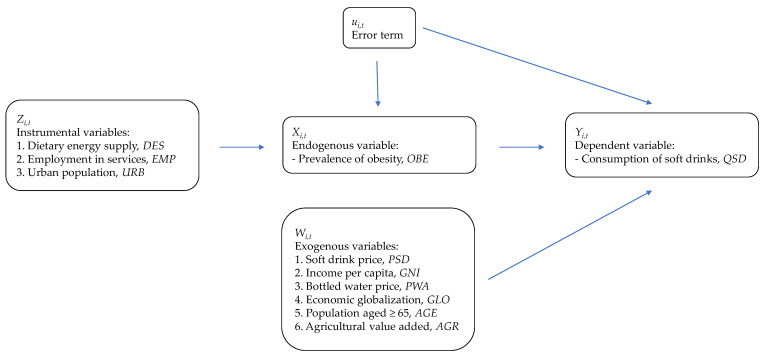
Overview of the regression model. To assess the impact of obesity on the consumption of soft drinks, in the first stage, we regressed the prevalence of obesity (*OBE*) on the instrumental variables *Z* and the set of exogenous variables *W*. The predicted value of *OBE* (denoted $\hat{OBE}$) resulting from the first stage was thus used in the second stage to estimate the demand for soft drinks.

Finally, in most countries, carbonated beverages are usually the most commonly consumed types of soft drinks. To further explore the role of obesity in determining the demand for this type of beverages, the following demand model for the carbonated soft drinks was estimated using the same above-described methodology:

(2) $${\mathrm{QCA}}_{\mathrm{it}}=\beta_{0}+\beta_{1}{\hat{OBE}}_{\mathrm{it}}+\beta_{2}{\mathrm{PCA}}_{\mathrm{it}}+\beta_{3}{\mathrm{GNI}}_{\mathrm{it}}+\beta_{4}{\mathrm{PWA}}_{\mathrm{it}}+\beta_{5}{\mathrm{GLO}}_{\mathrm{it}}+\beta_{6}{\mathrm{AGE}}_{\mathrm{it}}+\beta_{7}{\mathrm{AGR}}_{\mathrm{it}}+\beta_{8}{\mathrm{PNC}}_{\mathrm{it}}+\alpha_{i}+u_{\mathrm{it}}$$

where *QCA* denotes the per capita consumption of carbonated soft drinks, and *PCA* and *PNC* are the average prices of carbonated and non-carbonated soft drinks, respectively, whereas the other variables have the usual meanings. An overview of the regression models is shown in Figure A1 in Appendix B.

## 3. Results

The results obtained from the 2SLS regression analysis, as reported in Table 2, suggest that a significant direct relationship exists between the prevalence of obesity and the consumption of soft drinks. The left-hand side of Table 2 summarizes the regression results based on equation 1), in which the dependent variable is *QSD* (i.e., the consumption of all types of soft drinks). Overall, all explanatory variables were statistically significant at *p* < 0.01 and displayed the expected sign. Changes in the obesity rate were associated with changes in soft drink consumption. Specifically, holding fixed all other factors affecting *QSD*, a one-unit increase in the prevalence of obesity (i.e., a one percent increase in the age-adjusted rate of obesity) increased the consumption of soft drinks by about 2.37 L per person/year.

Consumption also responded to changes in price and in the price of related goods, as expected. *QSD* decreased by around 2.87 L for each one-unit increase in the average price per liter. Conversely, a one-unit increase in the price of bottled water raised *QSD* by about 3 L, indicating that soft drinks and bottled (carbonated and still) water were substitutes in consumption. Moreover, the positive sign of the variable GNI denoted that soft drinks were normal goods, whose consumption increased with income (for each $1000 increase in income per capita, consumption increased by about 0.7 L). Finally, more economic globalization positively affected soft drink consumption, and increases in the share of elderly people and in the weight of the agricultural sector decreased consumption of soft drinks by approximately 5 and 0.3 L per person/year, respectively. The right-hand side of Table 2 presents the estimation results of the demand for carbonated soft drinks (Equation (2)). There are no big differences. Again, all explanatory variables were statistically significant at *p* < 0.01 and displayed the expected sign, except for the price of non-carbonated soft drinks (this was probably due to the inclusion in the category of non-carbonated soft drinks of products, such as energy/functional and sports drinks, as well as ready-to-drink coffee, whose consumption is not associated with that of typical sodas).

Finally, 2SLS regression analysis relies on the validity of the instrumental variables. As already stressed, a good instrumental variable must be relevant (i.e., a good proxy of the endogenous one) and exogenous (i.e., uncorrelated with the error term). The bottom lines of Table 2 also report the results of the diagnostic tests on the three instruments utilized (the variables *DES*, *EMP*, and *URB*). Specifically, the underidentification test refers to the ‘relevance’ of the instruments by determining whether the variation in the instruments is related to the variation in the endogenous variable (i.e., the prevalence of obesity). Instead, the Sargan–Hansen J-statistic deals with the exogeneity of the instrumental variables. This test is used to check whether the instruments are correlated with the estimated residuals. In other words, it tests if that part of the variation of the endogenous variable captured by the instrumental variable is exogenous [34].

Regarding the relevance of the instruments, we rejected the null hypothesis that the regression models were underidentified (*p*-values < 0.001). This result indicates that the instruments chosen were relevant. We also found a first-stage F-statistic larger than Stock–Yogo critical values (i.e., weak identification test), suggesting that our instruments were not weak. On the other hand, the *p*-values of the J-statistic for the overidentification test of all instruments for Equations (1) and (2) were 0.72 and 0.11, respectively. These values indicate that we cannot reject the null hypothesis that the instruments were valid (i.e., uncorrelated with the error term in the second stage).

## 4. Discussion

These results indicate the existence of a feedback effect of the prevalence of obesity on the consumption of soft drinks. For instance, a one-unit increase in the prevalence of obesity was associated with a higher carbonated soft drink consumption of approximately 1.11 L. This implies that slightly more than one-third of the increase in soft drink consumption due to the increased prevalence of obesity was directed towards carbonated soft drinks. The prevalence of obesity should thus be included among the determinants of the demand for soft drinks.

**Figure 2:**
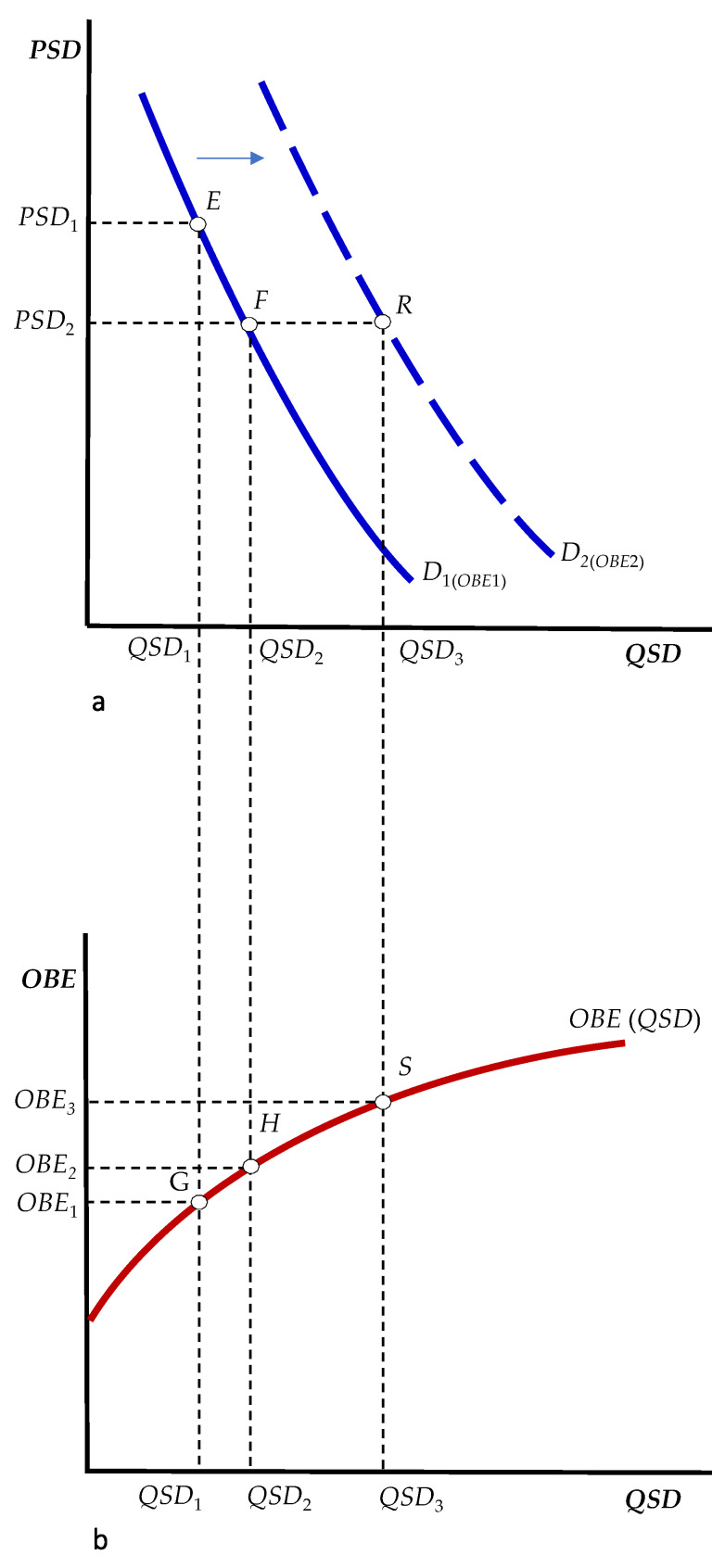
(**a**) The impact of the prevalence of obesity on the market demand for soft drinks, (**b**) The impact of soft drink consumption on the prevalence of obesity. Note: *QSD*, soft drink consumption per capita (liters/person/year); *PSD*, soft drink price (average price per liter, constant 2017 international $); *OBE*, prevalence of obesity (BMI ≥ 30 kg/m^2^. Age-stand. rate, both sexes, 18+ years, %).

Understanding the potential impact of the spread of obesity on the market demand for soft drinks allows policy maker to design a comprehensive strategy to tackle the spread of unhealthy eating habits. Figure 2a,b illustrate the implications for public health of our results. The curve labeled *D*_1_ depicted in Figure 2a shows the quantity of soft drinks (*QSD*) consumers are willing to buy at a given price (*PSD*), holding constant any other factors that might affect the quantity demanded. Specifically, *D*_1_ is depicted for a given obesity rate (*OBE*_1_) in the population under study. The bivariate relationship between soft drinks and the prevalence of obesity is shown in Figure 2b, where the prevalence of obesity (*OBE*, on the y-axis) increases with soft drinks consumption (*QSD*, on the x-axis).

At price *PSD*_1_, the quantity demanded is *QSD*_1_ (point *E* in Figure 2a), implying a prevalence rate of obesity equal to *OBE*_1_ (point *G* in Figure 2b). A decrease in price from *PSD*_1_ to *PSD*_2_—due, for instance, to an aggressive pricing strategy which reflects greater competition among producers and/or distributors—leads to an increase in the quantity demanded to *QSD*_2_ (i.e., a movement along the *D*_1_ curve until point *F*), which results in a greater prevalence of obesity (*OBE*_2_, point *H* in Figure 2b). However, *OBE*_2_ is not the final equilibrium because this greater spread of obesity increases the demand for soft drinks (i.e., the entire demand curve shifts to the right, from *D*_1_ to *D*_2_ in Figure 2a). That is, at *OBE*_2_ corresponds the dashed demand curve *D*_2_, reflecting a rise in the quantity demanded at any given price. As a result, the quantity demanded at the aggressive low-price strategy *PSD*_2_ is no longer *QSD*_2_, but it increases to *QSD*_3_ (point *R* on the *D*_2_ demand curve), which, in turn, increases the prevalence of obesity to *OBE*_3_ (point *S*, in Figure 2b), and so forth.

In other words, the initial decrease in the price of soft drinks has both a direct and an indirect effect on the prevalence of obesity. The direct effect (from *OBE*_1_ to *OBE*_2_) is due to the increase in the quantity demanded (i.e., the downward movement from *E* to *F* along the initial demand curve *D*_1_). The indirect effect (from *OBE*_2_ to *OBE*_3_) is due to the increase in demand (i.e., the rightward shift of the entire demand curve from *D*_1_ to *D*_2_) caused by the increased prevalence of obesity.

### Limitations

Several limitations should be acknowledged when interpreting these results. Firstly, the soft drink industry is targeting low-income consumers in both advanced and emerging economies. Our dataset includes only three low-income countries, and we do not account for income distribution and inequalities within countries. Secondly, our results must be interpreted considering that consumption and obesity data relate to the total and adult populations, respectively. Because of the popularity of soft drinks among children and adolescents, the impact of obesity on consumption should be assessed by separating the population into age groups (e.g., ≤18 and >18 years old), provided that suitable data are available.

Thirdly, there are notable differences in the quantity of sugar contained in the different kinds of soft drinks [37]. For instance, some regular sodas contain less than 10 g of sugar per eight oz. serving, and others more than 45 g (approximately 2.4 and 10.7 teaspoons of table sugar, respectively). Due to the use of aggregate market data, we cannot capture these differences between beverage categories. A fourth limitation is the obvious source of reverse causality between soft drinks and obesity that comes from individuals living with obesity or metabolic risk who are more likely to consume artificially sweetened beverages (ASBs) to control weight. However, most of the time, these individuals are already soft drink consumers who merely switch from regular (i.e., with added sugar) to diet beverages, leaving unchanged the overall impact of obesity on soft drink consumption.

Furthermore, during recent years a number of countries worldwide have implemented promising measures to prevent obesity, such as fiscal policies (e.g., taxation and subsidies), regulatory policies (e.g., bans and standards on the food and beverage industry marketing and advertising strategies), and nutrition education programs [38,39]. These policies are potential confounding factors that we do not include in the study due to a lack of reliable data. Finally, another limitation of the analysis could be the use of a country fixed effects model, which comes at the cost of less precise estimations than the random-effects model [40]. However, as random effects models have more stringent assumptions, we preferred to be more conservative and carefully controlled for country fixed effects. All these potential limitations come especially from the use of aggregate market data. Further research should be undertaken to investigate the impact of the prevalence of obesity on the demand for soft drinks with micro (i.e., individual) data, using a broader range of control variables and different types of ultra-processed foods and beverages.

## 5. Conclusions

This study was designed to determine whether the prevalence of obesity should be included among the factors affecting the market demand for soft drinks. The evidence indicates that changes in the age-adjusted obesity rate shift the demand curve and results in a feedback effect of obesity on soft drink consumption. This interplay between the consumption of soft drinks and the prevalence of obesity corroborates the idea that the development of an obesogenic food environment is a dynamic self-sustaining process [7], in which obesity and unhealthy lifestyles tend to reinforce each other (i.e., increases in obesity rates, due to the consumption of soft drinks, promote additional increases in the demand for soft drinks). The result is a vicious cycle that fosters the spread of obesity. This evidence further supports the need for an integrated approach to curb soft drink consumption, wherein fiscal tools (i.e., ‘sugar taxes’) must be combined with other measures (such as bans, regulations, and nutrition education programs) in order to exploit this feedback mechanism in the opposite direction, triggering a virtuous cycle of decreasing demand for soft drinks and obesity rates.

## Figures and Tables

**Table 1:** Summary of variables and descriptive statistics.

Variable	Description	Mean	Std. Dev.	Min	Max	N. of Obs.
*QSD*	Soft drink consumption per capita (liters/person/year)	75.38	49.00	1.69	270.95	1485
*OBE*	Prevalence of obesity (BMI ≥ 30 kg/m^2^. Age-stand. rate, both sexes, 18+ years, %)	18.62	8.78	0.90	39.70	1.455
*PSD*	Soft drink price (average per liter PPP, constant 2017 international $)	3.44	1.23	1.58	15.12	1470
*GNI*	Gross national income per capita (PPP, constant 2017 international $)	24,410.07	20,070.88	892.83	97,094.19	1470
*PWA*	Bottled still and carbonated water price (average per liter PPP, constant 2017 international $)	1.73	0.75	0.30	5.58	1470
*GLO*	KOF index of economic globalization (min = 0, max = 100)	63.17	15.73	25.5	95.3	1470
*AGE*	Population aged 65 and above (as % of total population)	10.17	6.16	0.69	28.00	1470
*AGR*	Agricultural value added (as % of total value added, GDP)	7.97	8.37	0.03	46.69	1470
*DES*	Dietary energy supply (kcal/person/day)	2989.13	422.89	1729	3847	1485
*EMP*	Employment in services (both sexes, as % of total employment)	59.27	16.79	14.76	88.29	1470
*URB*	Urban population (as % of total population)	65.36	20.27	15.70	100.00	1470
*QCA*	Carbonated soft drink consumption per capita (liters/person/year)	49.70	35.70	1.38	186.89	1485
*PCA*	Carbonated soft drink price (average per liter PPP, constant 2017 international $)	3.03	1.14	1.26	12.38	1470
*PNC*	Non-carbonated soft drink price (average per liter PPP, constant 2017 international $)	4.64	2.64	1.80	35.57	1470

Notes: BMI: Body mass index; PPP: Purchasing power parity; KOF: Swiss Economic Institute; GDP: Gross Domestic Product.

**Table 2:** Instrumental variable (IV) regression results: assessing the impact of the prevalence of obesity on the demand for soft drinks.

Dependent Variable							
Soft Drink Consumption, *QSD*				Carbonated Soft Drink Consumption, *QCA*			
Independent Variables		Coefficient	Std. Error ^1^	Independent Variables		Coefficient	Std. Error ^1^
Prevalence of obesity	*OBE*	2.3660 ***	0.2267	Prevalence of obesity	*OBE*	1.1150 ***	0.1574
Soft drink price	*PSD*	−2.8759 ***	0.8378	Carbonated soft drink price	*PCA*	−3.0533 ***	0.7151
Income per capita	*GNI*	0.0007 ***	0.0001	Income per capita	*GNI*	0.0003 ***	0.0001
Bottled water price	*PWA*	3.0471 ***	1.0276	Bottled water price	*PWA*	2.7238 ***	0.8639
Economic globalization	*GLO*	0.3393 ***	0.0908	Economic globalization	*GLO*	0.2706 ***	0.0734
Population aged 65 and above	*AGE*	−5.0116 ***	0.4848	Population aged 65 and above	*AGE*	−3.4424 ***	0.3390
Agricultural value added	*AGR*	−0.3045 ***	0.0903	Agricultural value added	*AGR*	−0.2515 ***	0.0590
				Non-carbonated soft drink price	*PNC*	0.0466	0.1844
N. of obs.	1455			N. of obs.	1455		
F-statistic, F(7, 1351)	39.62, Prob. 0.000			F-statistic, F(8, 1350)	21.77, Prob. 0.000		
Underidentification test	206.808, P-val. 0.000			Underidentification test	197.131, P-val. 0.000		
Weak identification test	327.346			Weak identification test	323.315		
Sargan–Hansen J statistic	0.668, P-val. 0.716			Sargan–Hansen J statistic	4.379, P-val. 0.112		

Notes: ^1^ Heteroskedasticity-robust standard errors. *** Denotes statistically significant correlation at the 0.01 probability levels (2-tailed). In both regression equations, two-stage least square estimation with fixed effects (Instrumented: Prevalence of obesity, *OBE*. Instruments: Dietary energy supply, *DES*. Employment in services, *EMP*; Urban population, *URB*). Underidentification test (Kleibergen–Paap rk LM statistic), weak identification test (Cragg–Donald Wald F statistic), Sargan–Hansen J statistic (overidentification test of all instruments).

## Data Availability

The dataset is available at Mendeley Data repository (https://doi.org/10.17632/hkm25rbpsc.2, accessed on 20 October 2020).

## Supplementary Materials

The following supporting information can be downloaded at: https://www.mdpi.com/article/10.3390/ijerph19020938/s1. We used secondary data. All data generated or analyzed during this study are included in this published article and in its Supporting Information File: File S1—Dataset.

## Appendix A

**Table A1:** List of countries included in the study by income group.

**High-Income Countries**
Australia (WPA), Austria (EUR), Belgium (EUR), Canada, (AME), Chile (AME), Croatia (EUR), Czech Republic (EUR), Denmark (EUR), Estonia (EUR), Finland (EUR), France (EUR), Germany (EUR), Greece (EUR), Hong Kong (WPA), Hungary (EUR), Ireland (EUR), Israel (EME), Italy (EUR), Japan (WPA), Kuwait (EME), Latvia (EUR), Lithuania (EUR), Netherlands (EUR), New Zealand (WPA), Norway (EUR), Oman (EME), Panama (AME), Poland (EUR), Portugal (EUR), Qatar (EME), Saudi Arabia (EME), Singapore (WPA), Slovakia (EUR), Slovenia (EUR), South Korea (WPA), Spain (EUR), Sweden (EUR), Switzerland (EUR), Taiwan (WPA), United Arab Emirates (EME), United Kingdom (EUR), Uruguay (AME), United States of America (AME). No. = 43
**Upper-Middle Income Countries**
Algeria (AFR), Argentina (AME), Azerbaijan (EUR), Belarus (EUR), Bosnia and Herzegovina (EUR), Brazil (AME), Bulgaria (EUR), China (WPA), Colombia (AME), Costa Rica (AME), Dominican Republic (AME), Ecuador (AME), Georgia (EUR), Guatemala (AME), Iraq (EME), Jordan (EME), Kazakhstan (EUR), Lebanon (EME), Malaysia (WPA), Mexico (AME), North Macedonia (EUR), Paraguay (AME), Peru (AME), Romania (EUR), Russia (EUR), Serbia (EUR), South Africa (AFR), Sri Lanka (SEA), Thailand (SEA), Turkey (EUR). No. = 30
**Lower-Middle Income Countries**
Angola (AFR), Bangladesh (SEA), Bolivia (AME), Cambodia (WPA), Cameroon (AFR), Côte d’Ivoire (AFR), Egypt (EME), El Salvador (AME), Ghana (AFR), Honduras (AME), India (SEA), Indonesia (SEA), Kenya (AFR), Laos (WPA), Morocco (EME), Myanmar (SEA), Nigeria (AFR), Pakistan (EME), Philippines (WPA), Tunisia (EME), Ukraine (EUR), Uzbekistan (EUR), Vietnam (WPA). No. = 23
**Low-Income Countries**
Ethiopia (AFR), Tanzania (AFR), Uganda (AFR). No. = 3

Notes: World Bank country classifications by income level. GNI per capita 2019 in current USD: Low income < 1036; Lower-middle income 1036–4045; Upper-middle income 4046–12,535; High income > 12,535. WHO Regions: AFR African, EUR European, EME Eastern Mediterranean, AME Americas, SEA South-East Asia, WPA Western Pacific. Honk-Kong and Taiwan were excluded from the regression analysis.

## Appendix B

A simplified IV regression model may be written as:

*Y_i_* = *β*_0_ + *β*_1_*X_i_* + *β*_2_*W*_1*i*_ + … + *β_k_W_ki_* + *u_i_* (A1)

where *Y* denotes the dependent variable, *X* is the endogenous regressor, *W* represents the list of exogenous variables, and *u* is the error term. If *X* and *u* are correlated, the standard OLS estimator is biased due to the simultaneous causality between *X* and *Y*. The 2SLS overcomes this problem by applying two OLS separated regressions: the first one, to obtain the predicted value of the endogenous variable ($\hat{X}$), by regressing *X* on one or more suitable instrumental variables and the exogenous variables *W*; the second one, to estimate the impact of *X* on *Y* (i.e., to obtain an unbiased estimation of the coefficient *β*_1_ in Equation (A1)) by regressing *Y* on the predicted value of *X* ($\hat{X}$) and on the set of exogenous variables *W*. In our empirical model, as shown by the causal pathway depicted in Figure A1, there were three instrumental variables (*Z*)—i.e., (1) the dietary energy supply (*DES*), (2) the share of employment in the services sector (*EMP*), and (3) the degree of urbanization (*URB*)—and a set of six exogenous variables (*W*), namely: (1) the price of soft drinks (*PSD*), (2) the price of bottled water (*PWA*), (3) the income per capita (GNI), (4) the degree of globalization (*GLO*), (5) the share of the total population aged 65 or more (*AGE*), and (6) the share of agricultural value-added on the country total value added (*AGR*).
